# The First Case Report of a Primary Mast Cell Tumor Originating from the Inguinal Lymph Node in a Nine-Year-Old Female Maltese Dog and a Comparative Literature Review in Humans

**DOI:** 10.3390/life15071029

**Published:** 2025-06-27

**Authors:** Nuri Lee, Gibum Kwon, Kyuhyung Choi

**Affiliations:** 1 Yeeun Animal Hospital, Seoul 06052, Republic of Korea; inuri1991@yonsei.ac.kr (N.L.); sdvm@naver.com (G.K.); 2 Department of Medical Device Engineering and Management, College of Medicine, Yonsei University, Seoul 03722, Republic of Korea; 3 Transdisciplinary Department of Medicine & Advanced Technology, Seoul National University Hospital, Seoul 03080, Republic of Korea; 4 Bundang New York Animal Hospital, Seongnam 13637, Republic of Korea

**Keywords:** mast cell tumor, lymph node, mammary gland tumor

## Abstract

Here, the authors report the first case of a primary mast cell tumor originating from the inguinal lymph node in a nine-year-old intact female Maltese dog that had undergone a left ureteral stent, ureterotomy and splenectomy, and left-side mastectomy, including inguinal lymph node removal and ovariohysterectomy, in South Korea in May 2024. The splenic mass, mammary gland mass, and inguinal lymph node underwent histopathological examination, resulting in the diagnosis of nodular hyperplasia, grade 1 mammary complex carcinoma, and a mast cell tumor (MCT), respectively. To clarify the origin of the MCT from the inguinal lymph node, a computed tomography (CT) scan was performed. In addition, through a blood smear test, mast cell leukemia was ruled out. After CT scanning by veterinary radiologists and a biopsy of all possible masses, it was finally concluded that the MCT primarily originated from lymph nodes, which is extremely rare in dogs. The patient is recovering well as of February 2025, just 7 months after the first diagnosis, following surgery and 16 weeks of chemotherapy with a combination of prednisolone and vinblastine, considering the C-kit PCR results of the left inguinal lymph node after the surgical removal of the MCT. This report is significant for two reasons, firstly because of the rarity of MCTs originating from lymph nodes other than the skin and gastrointestinal organs, and secondly because the authors propose a hypothesis for the rarity of primary lymph node mast cell tumors and the correlation between mammary gland tumors and mast cell tumor growth based on a comparative literature review in humans, focusing on molecular mechanisms.

## 1. Introduction

Mast cells are bone-marrow-derived cells that are involved in allergic and hypersensitivity reactions and also secrete histamine, heparin, and proteases [1]. Mast cell tumors (MCTs) account for 21% of common dog skin cancers [2], and boxers (1.95%) and golden retrievers (1.39%) are commonly known to develop MCTs [3]. The metastasis of an MCT to another organ is approximately 4% [4]. In dogs, MCTs mostly occur on the skin [5], but they can also occur in other organs, such as the gastrointestinal tract [6], oral mucosa [7], and lungs [8]. Primary mast cell tumors originating from lymph nodes are exceedingly rare, with only a few reports having documented cases in visceral lymph nodes such as the hepatopancreatic lymph node [9] and the mesenteric lymph node [10,11]. To date, there have been no documented cases of primary mast cell tumors originating from cutaneous lymph nodes in dogs. Here, the authors report the first canine case of a primary mast cell tumor originating from the inguinal lymph node. Additionally, in humans, mast cells are associated with various types of tumors, either contributing to their inhibition or promoting their growth [12]. Mast cells are hypothesized to contribute to the development of canine mammary malignant tumors (MGTs) by promoting angiogenesis, similar to some tumors described in humans [13]. The role of MCTs in promoting MGT progression is not fully elucidated, although evidence from human studies suggests that mast cells play a role in weakening stromal–epithelial interactions, and this process may contribute to MGT development and facilitate angiogenesis [12]. The case in this study also supports this point, as the MCT primarily originated from the nearby inguinal lymph node and facilitated the concurrent growth of the MGT. We also investigate the molecular mechanism of correlation between the MCT and MGT through a literature review and provide a plausible explanation for this case by focusing on vascular endothelial growth factor (VEGF) [14] and tumor-associated macrophages (TAMs) [15]. Additionally, we explore the possibility of utilizing single-cell techniques in this case report and for further studies.

## 2. Case Presentation

On 16 March 2024, a splenic tumor and kidney stones were incidentally found in a 9-year-old intact female Maltese visiting a local hospital and were referred to Yeeun Animal Hospital (Seoul, Republic of Korea). After physical examinations in Yeeun Animal Hospital, mammary gland masses were additionally found through palpation, which were located in the left third and fifth mammary glands. On 1 May, fine-needle aspiration (FNA) was performed in the left third and fifth mammary glands. After checking the mast cell tumor in the left fifth mammary gland using FNA and the benign tumor in the left third mammary gland, efforts to find a clear primary mast cell tumor origin, including additional physical examinations, radiography, and abdominal ultrasonography, were performed on 4 May.

**Figure 1:**
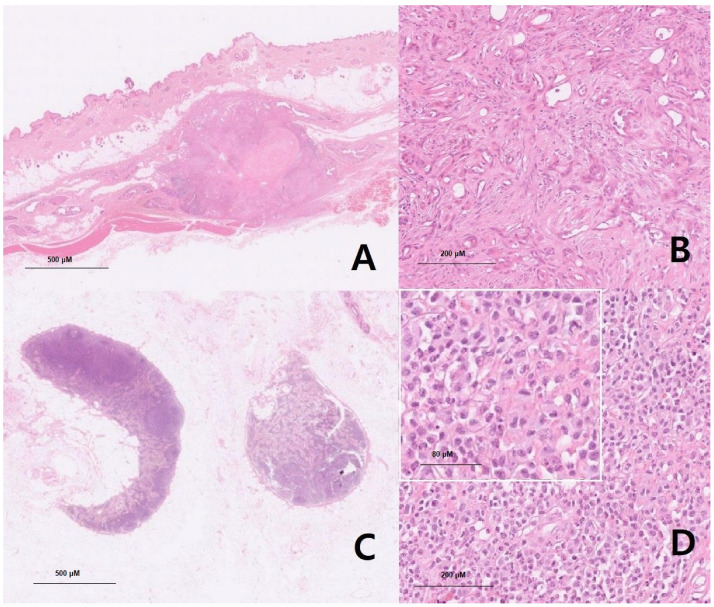
(**A**–**D**) Histopathologic examination of the masses (mammary gland and inguinal lymph node).

**Figure 2:**
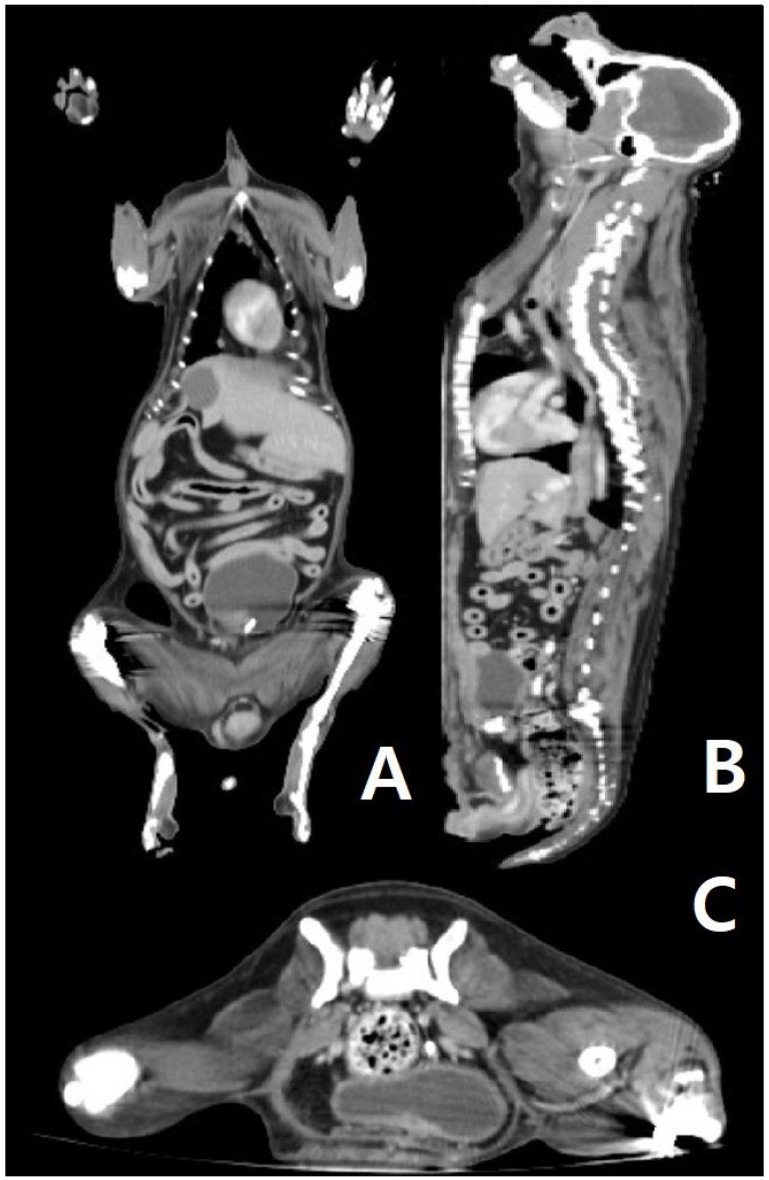
(**A**–**C**): CT scan of the patient’s body. (**A**) In the dorsal view of the CT scan, there is no sign of a primary tumor in the abdominal organ and thorax. (**B**) In the sagittal view of the CT scan, there is no sign of a primary tumor in the abdominal organ and thorax. (**C**) In the transverse view of the CT scan (posterior abdomen), there is no sign of a primary tumor in the abdominal organ and thorax.

After the examination and procedures, hydronephrosis arose due to a ureteral stone descending from the left kidney. Therefore, emergency surgery was performed on 8 May with comprehensive surgical procedures, including a ureterotomy for the ureteral stent, stone removal, a left partial mastectomy with the inguinal lymph node, a splenectomy, and an ovariohysterectomy. In addition, a histopathological examination (Figure 1) was performed by Antech (Seongnam, Republic of Korea) after the comprehensive surgery, and the results showed nodular lymphoid hyperplasia (spleen), a grade 1 mammary complex carcinoma (left third mammary tumor), and a mast cell tumor (left inguinal lymph node), respectively, on 23 May, as shown in Table 1. An additional C-kit mutation PCR test was performed by the Michigan State University Veterinary Diagnostic Laboratory (Lansing, MI, USA) in June 2024. At the pet owner’s request, IHC staining (immuno-histochemical staining) was not performed, although it is an important tool for the confirmation of tumor origin. Additionally, mast cell leukemia was ruled out via a peripheral blood smear. Finally, CT scans were performed to find the mast cell tumor’s origin on 21 June 2024 at Shine Animal Hospital (Seoul, Republic of Korea). After the CT scan, an additional biopsy of the mast cell tumor origin candidates was performed by Antech, and three inguinal nodules were diagnosed as mild lymphoid follicular hyperplasia, while the other three masses found in the hind body were diagnosed as nodular sebaceous hyperplasia, lipoma, and normal-haired skin, respectively, on 27 June 2024 (Table 1). As mast cells are generally derived from the bone marrow, veterinary radiologists thoroughly analyzed the tumor findings of the bone marrow from head to toe, utilizing GE Healthcare Revolution ACT (IL, USA), and found no remarkable findings in the bone marrow (Figure 2).

**Figure A2:**
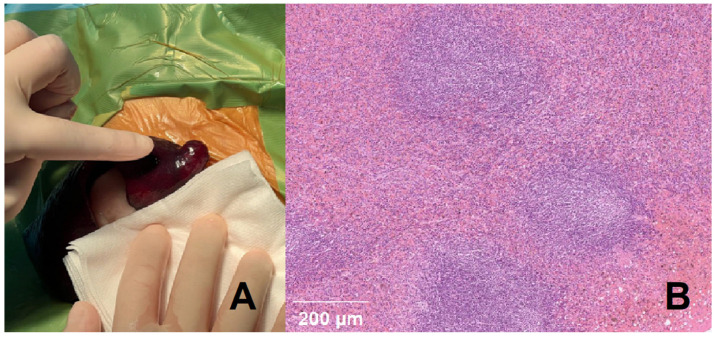
(**A**) Macroscopic lesion of the splenic mass. (**B**) Nodular lymphoid hyperplasia of the spleen. Sections of the spleen contained a large nodular mass that was characterized by the proliferation of multiple hyperplastic lymphoid follicles surrounded by red blood cells admixed with hematopoietic precursor cells and megakaryocytes. Lymphoid follicles had retained polarity and prominent mantle zones. There was no evidence of neoplastic disease within the sections examined histologically. (Scale bars = 200 μM, inset).

Based on all imaging tests and biopsies that were performed on this dog, the tumor was diagnosed as a primary mast cell tumor that originated from the left inguinal lymph node. Even though the findings of histopathological examinations, such as those shown in Figure 1D, are generally seen in metastatic mast cell tumors, they may also be seen in primary mast cell tumors. There were no other findings of primary mast cell tumors in other organs (Appendix A, Figure A2), such as those shown in Table 1, as confirmed through consecutive biopsy and CT scans (Figure 2).

After finding the origin of the tumor, the patient received vinblastine- and prednisolone-based chemotherapy for 16 weeks as C-kit PCR 8 and 11 were negative (Table 2), which can be interpreted as being less likely to respond to tyrosine kinase inhibitors. The chemotherapy protocol was started on 20 July, and vinblastine was administered intravenously at a dose of 2 mg/m^2^ biweekly for a total of eight treatments (16 weeks). Prednisolone (2 mg/kg) was orally administered daily and was gradually tapered before discontinuation. As of October 2024, no metastases, recurrences, or new tumors were observed in follow-up examinations. As of February 2025, the patient is recovering well with no clinical signs or tumor recurrence to date, as well as in April 2025.

Specimens (A) and (B): Left third mammary gland mass, 0.8 cm × 0.6 cm (4.4 cm × 3.2 cm including skin), firm and solid with no hemorrhage, and solitary.

Microscopic description: (A) At ×40 magnification, neoplastic epithelial cells were surrounded by myoepithelial cells and were well-demarcated and nodular. Anisocytosis and anisokaryosis were moderate in the neoplastic cells in the mammary mass specimen, with no mitotic figures observed. (B) At ×100 magnification, myoepithelial cells appeared to be spindle-shaped to stellate, with indistinct cell borders and moderate amounts of amphophilic to eosinophilic to basophilic vacuolated to fibrillary to homogeneous cytoplasm and ovoid to elongate open nuclei with variable amounts of coarse chromatin and either indistinct or 1–2 small nucleoli. Neoplastic cells were polygonal to cuboidal and had variable distinct cell borders. Necrosis was seen in the central area, while numerous plasma cells and lymphocytes were present in the periphery. Grade 1 mammary complex carcinoma was confirmed comprehensively. Scale bars = 500 μM and 200 μM (inset).

Specimens (C) and (D): Left inguinal lymph node, 1.1 cm × 1.0 cm, firm and solid with no hemorrhage, and solitary.

Microscopic description: (C) At ×40 magnification, moderate follicular and paracortical hyperplasia were observed in the lymph node specimen, accompanied by moderate sinus histiocytosis. Anisocytosis and anisokaryosis were moderate, and mitotic figures were frequently observed. (D) At ×100 magnification, the neoplastic mast cells featured distinct cell borders and exhibited a round morphology with moderate amounts of cytoplasm containing basophilic granules. Finely stippled chromatin was centrally located in an oval nucleus. A small number of eosinophils were mixed among neoplastic mast cells. These effacements of normal nodal structure, along with an overt number of mast cells, are generally seen in metastatic mast cell tumors (HN3, scale HN0–HN3). Scale bars = 500 μM, 200 μM, and 80 μM (inset).

## 3. Discussion

Canine MCTs primarily occur in the skin, as previously described, with numerous cases reported of cutaneous MCTs metastasizing to other organs, such as the heart [16], bone marrow [17], urinary bladder, spleen, liver, and kidney [18]. The involvement of lymph nodes is common, and identifying the primary organ of MCTs is essential for prognosis and treatment. [18].

During the process of finding the origin of the MCT, mast cell leukemia, which is rare in dogs [19], was first considered due to the infiltration of mast cells in the lymph nodes. However, this was excluded since there was no increase in the number of mast cells in a peripheral blood smear. During the first surgery, the large tumor on the left mammary gland and the spleen was removed, and these organs were found to not be a primary MCT. After the surgery to clarify the primary organ of the MCT, even the small nodules found through CT were biopsied, but no suspected mast cell tumors other than the lymph nodes were found. Therefore, the patient in this case was confirmed as having a primary MCT of the lymph node, and it is significant as this is the first case to utilize ultrasound, CT scans, and biopsies to scrutinize the origin.

In general, a canine lymph node primary MCT is uncommon, and to the authors’ knowledge, there have been no case reports since 2009 [11]. Previous reports of primary MCTs in lymph nodes are rare and include cases in the hepatopancreatic lymph node [9], cranial mesenteric lymph node [10], and mesenteric lymph node [11]. This is the first reported case of an MCT primarily occurring in cutaneous lymph nodes and is notable for its academic significance in employing advanced imaging techniques for the identification of a primary cutaneous MCT and performing biopsies on all suspicious lesions. Cutaneous lymph nodes have the advantage of being easily palpated during physical examination and being accessible for cytological evaluation. If mast cell infiltration is observed in lymph nodes with a concurrent MCT in a nearby skin mass, which can be confirmed by cytological examination, it is generally considered to be lymph node metastasis from proximal skin tumors. However, as shown in this case, if a primary mass is not identified near the lymph nodes via physical examination or imaging, the possibility of a primary MCT in the lymph nodes should be considered.

In addition, this patient did not show any lymph node changes upon physical examination as of December 2024, after the first diagnosis of a primary MCT of the lymph nodes in March 2024, and other skin masses are not palpable. In addition, X-ray U/S phase metastasis has not been confirmed. This is also believed to enhance the academic value of this paper by providing prognostic information for primary surface lymph node metastatic cancer (MCT) treatment involving surgical removal and adjuvant chemotherapy through continuous follow-up monitoring. Since there was no C-kit mutation in the specimen, as confirmed by PCR testing, chemotherapy was performed as opposed to targeted therapy according to the standard protocol [20,21].

### 3.1. Correlation Between MCT and MGT Growth

Although it is uncertain whether an MGT is positively correlated with a primary MCT, and in some case reports, MCTs and MGTs arise concurrently [22], MGTs are common in senior intact female dogs; therefore, the occurrence of an MCT and an MGT in the patient may be an independent event. Additionally, the BRCA-1 gene mutation, which is crucial in breast cancer, affects the regulation of VEGF, which leads to the dysregulation of the gene and increases angiogenesis in humans [23]. In addition, mast cells release VEGF and promote tumor angiogenesis in humans [24], and a similar phenomenon was detected in canines [25], although the detailed mechanism has not been as well-documented in canines as in humans. Particularly, VEGF-B is overexpressed in MGTs in canines [26], and activation of mast cells during lymphangiogenesis leads to VEGF-B secretion in humans [27].

On the other hand, there are subclasses of macrophages: M0 [28], M1, M2, and M2-like TAM [29]. MGTs and TAM are positively correlated in canines [30], and TAM also releases cytokines such as VEGF and TNF-α. TAM and mast cells work together in the tumor environment, inhibiting or facilitating the progression of the tumor depending on its stage [31]. It has also been reported that tumor-associated mast cells are less frequently observed in malignant mammary gland tumors than in mammary gland hyperplasia [32]. Considering these factors together, it is not clear whether the growth of MCTs and MGTs is positively correlated, as this is multifactorial.

### 3.2. Hypothesis of a Rare Primary Lymph Node MCT

Mast cells release VEGF [33], and the overexpression of VEGF in the skin leads to the growth of tumors [34]. Therefore, by putting these facts together, it is plausible to determine that a cutaneous MCT is more common than in other organs. On the other hand, the endothelium of lymphatic vessels is more permeable than the endothelium of blood vessels [35], making attachment more difficult in blood vessels and contributing to the tumor microenvironment [36]. However, mast cells lead to chemotaxis to laminin [37] and are essential in the metastasis of primary tumors, as mediated by macrophages [38]. Nevertheless, it is theoretically possible that MCTs primarily originate from the lymph node, as previously reported in the visceral lymph node [9], and this study is the first to report an MCT originating from the cutaneous lymph node.

### 3.3. Spatial Transcriptomics and Single-Cell Sequencing of the Lymph Node MCT

**Figure 3:**
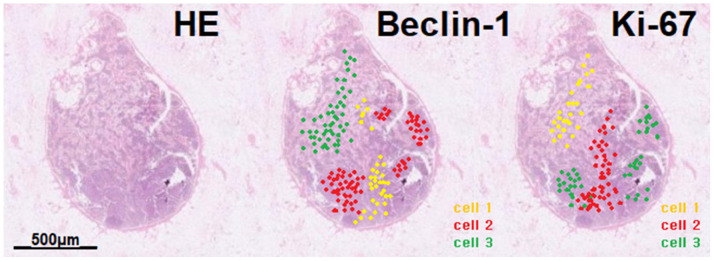
Schematic illustration of lymph spatial transcriptomics analysis regarding a mast cell tumor (yellow-, red-, and green-colored cell clusters).

With the advancement of single-cell sequencing [39], it is now possible to visualize protein expression at the single-cell level within a tissue slide [40]. If we can investigate the differentially expressed gene (DEG) regarding mast cell tumors, such as CD 117, Beclin-1, and Ki-67 in the primary lymph node tissue, and visualize the spatial RNA transcription at the single-cell level (Figure 3) using 10× visium and Seurat package in R, it will provide a deeper understanding of the development of mast cell tumors and the reason for their rarity. In addition, utilizing single-cell sequencing may lead to unexpected results regarding tumor angiogenesis and progression in canine mast cell tumors because there are relatively few documents that implement single-cell techniques in the veterinary medicine field compared to human medicine.

## 4. Conclusions

This case report is significant because it is the first to report that MCTs primarily originate from the inguinal lymph node by utilizing ultrasound, CT scans, and biopsies. We also provide a possible explanation for the rarity of MCTs originating from lymph nodes at the molecular level, which can be investigated in future follow-up research. Additionally, since there have been very few cases of primary MCTs in lymph nodes, no histopathological criteria currently exist for assessing prognosis. This case study also suggests that if numerous cases of lymph node primary MCTs are reported, pathological criteria for prognosis evaluation can be examined in a follow-up study.

## Figures and Tables

**Table 1:** Primary tumor candidates for the mast cell tumor.

	Organ	Mammary Gland	Spleen	Left Inguinal Lymph Node	Mast Cell Leukemia	Others (Including Inguinal and Hind Body Regions)
Method						
Full CT scan		Not applicable	Not applicable	Not applicable	Not applicable	Three inguinal nodules and three masses were found
Biopsy (or histopathology) results		Mammary complex carcinoma, grade 1	Nodular lymphoid hyperplasia	Mast cell tumor	Not applicable	Mild lymphoid follicular hyperplasia, nodular sebaceous hyperplasia, lipoma, and normal-haired skin
Blood smear		Not applicable	Not applicable	Not applicable	Ruled out	Not applicable

**Table 2:** C-kit PCR tissue results (left inguinal lymph node).

Procedure	Result
C-kit PCR specimen	Formalin-Fixed Paraffin-Embedded Tissue, positive
C-kit PCR 8 K9 Only	Negative
C-kit PCR 11 K9 Only	Negative

**Figure A1:**
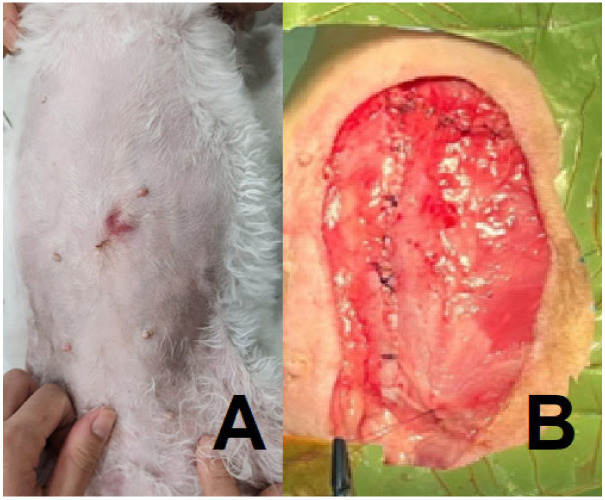
(**A**) Macroscopic lesion of the left inguinal lymph node mass. (**B**) Surgical removal of the left 3rd, 4th, and 5th mammary gland tumors.

**Figure A3:**
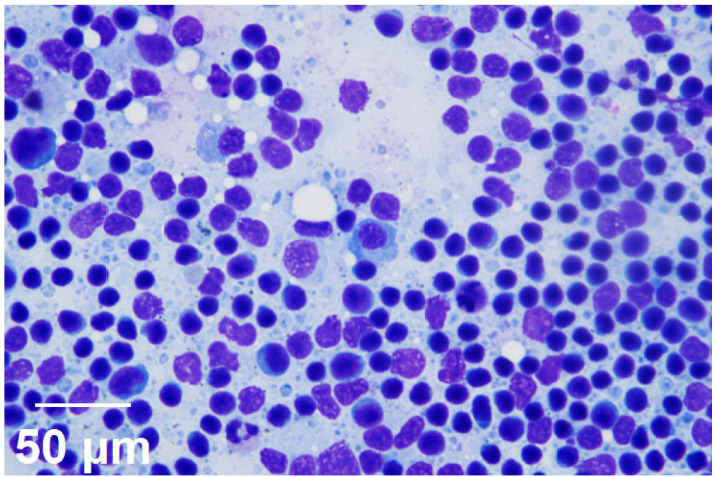
Reactive lymphoid hyperplasia (right inguinal lymph node) with moderate cellularity and mild erythrocyte infiltration. Most lymphocytes are small, with a small number of large lymphocytes and neutrophils. Macrophages and plasma cells are shown. There is no evidence of bacteria, parasites, or other inclusion bodies. (Scale bars = 50 μM, inset).

## Data Availability

The raw data supporting the conclusions of this article will be made available by the authors on request.

## Abbreviations

mast cell tumor (MCT); mammary gland tumor (MGT); tumor-associated macrophages (TAMs); vascular endothelial growth factor (VEGF)
